# Exhaled nitric oxide and airway hyperresponsiveness in workers: a preliminary study in lifeguards

**DOI:** 10.1186/1471-2466-9-53

**Published:** 2009-12-31

**Authors:** Valérie Demange, Abraham Bohadana, Nicole Massin, Pascal Wild

**Affiliations:** 1 INRS, Département Epidémiologie en Entreprise, Rue du Morvan, CS 60027, 54519 Vandœuvre-lès-Nancy Cedex, France; 2 INSERM, U954, Faculté de Médecine, 9, avenue de la forêt de Haye, 54500 Vandoeuvre- lès-Nancy, France; 3 Service de Pneumologie; CHU de Nancy; hôpital d'adultes de Brabois, avenue de Bourgogne, 54511 Vandoeuvre-lès-Nancy, France

## Abstract

**Background:**

Airway inflammation and airway hyperresponsiveness (AHR) are two characteristic features of asthma. Fractional exhaled nitric oxide (FENO) has shown good correlation with AHR in asthmatics. Less information is available about FENO as a marker of inflammation from work exposures. We thus examined the relation between FENO and AHR in lifeguards undergoing exposure to chloramines in indoor pools.

**Methods:**

39 lifeguards at six indoor pools were given a respiratory health questionnaire, FENO measurements, spirometry, and a methacholine bronchial challenge (MBC) test. Subjects were labeled MBC+ if the forced expiratory volume (FEV1) fell by 20% or more. The normalized linear dose-response slope (NDRS) was calculated as the percentage fall in FEV1 at the last dose divided by the total dose given. The relation between MBC and FENO was assessed using logistic regression adjusting on confounding factors. The association between NDRS and log-transformed values of FENO was tested in a multiple linear regression model.

**Results:**

The prevalence of lifeguards MBC+ was 37.5%. In reactors, the median FENO was 18.9 ppb (90% of the predicted value) vs. 12.5 ppb (73% predicted) in non-reactors. FENO values ≥ 60% of predicted values were 80% sensitive and 42% specific to identify subjects MBC+. In the logistic regression model no other factor had an effect on MBC after adjusting for FENO. In the linear regression model, NDRS was significantly predicted by log FENO.

**Conclusions:**

In lifeguards working in indoor swimming pools, elevated FENO levels are associated with increased airway responsiveness.

## Background

Airway inflammation is the hallmark of asthma[1]. Exposure to a variety of agents in the workplace can cause airway inflammation and occupational asthma. Thus, investigating airway inflammation from work exposure is important to elaborate preventive strategies.

Airway hyperresponsiveness (AHR) can be considered as a surrogate marker of airway inflammation and is recognized as another characteristic finding of asthma[1]. In the general population, AHR is a risk factor for an accelerated decline in forced expiratory volume in one second (FEV1) and for the development of asthma and chronic obstructive pulmonary disease[2]. In working populations, AHR is an important determinant for the development of symptoms[3].

Fractional concentration of exhaled nitric oxide (FENO) is another indirect marker of airway inflammation. FENO is increased in subjects with established asthma[4], is a more accurate detector of asthma than conventional tests[5], and has proved useful to monitor asthma treatment[6]. Moreover, the test is simple, quick to perform and has good reproducibility[7]. In working populations, elevated FENO levels have been found in non-smoking aluminium potroom workers[8], in underground construction workers[9], in shoe and leather workers[10], and in bleachery workers[11].

AHR correlates with FENO in steroid naïve asthmatics[12], adolescents in clinical remission of asthma[13], and atopic adults, asthmatics or not[14]. However, less is known about the relationship between AHR and FENO in workers undergoing exposure to pollutants in the workplace. Knowledge on this topic would help to better define the use of FENO as a tool in respiratory epidemiology.

The present study was carried out as a preliminary study to assess whether, in the context of occupation respiratory studies carried out in populations at work, the FENO could usefully complement the more traditional, but more difficult to use, methacholine bronchial challenge (MBC) test. This preliminary study was performed in a population of lifeguards working in indoor swimming pools, an occupation in which we had demonstrated a high prevalence of AHR in a previous study[15]. The purpose of the present paper is to show the feasibility of this approach and to examine the cross-sectional relationship between FENO and AHR as measured by the MBC test.

## Methods

The study was a cross-sectional survey of lifeguards from all six indoor swimming pools in an urban area of Eastern France. The examinations took place between April and June 2006 between 9:00 and 12:00 AM, or between 14:00 and 17:00 PM if morning examinations were not possible. The whole workforce was invited. Lifeguards with current asthma, not needing a corticosteroid treatment, and not in crisis, were included. All subjects gave written informed consent. The study was approved by the local medical ethics committee, Comité Consultatif de Protection des Personnes se prêtant à la Recherche Biomédicale de Lorraine, located in Nancy, France.

### Symptoms and smoking habits

A standard questionnaire indicated the past and present personal histories of cough, phlegm, physician-diagnosed asthma, wheezing, and dyspnea[15]. It included questions about work-related, irritant symptoms (ocular, nasal, respiratory) and a personal history of physician-diagnosed allergies (hay fever or eczema or urticaria). *Non-smokers *were defined as subjects who had never regularly smoked one or more cigarettes a day, or had smoked one or more cigarettes a day for less than one year. *Smokers *were defined as subjects who reported regular smoking of one or more cigarettes a day for at least one year. *Ex-smokers *were subjects who reported smoking one or more cigarettes a day regularly in the past but who had quit smoking at least one year prior to the study.

### FENO

FENO was measured using a chemiluminescence analyzer (Endono 8000, Seres, Aix en Provence, France) according to ATS/ERS recommendations[16]. The subject was in a sitting position and exhaled against an oral pressure of 5 cmH_2_0 -- sufficient to close the velum - at a constant flow of 50 mL.s^-1^; measurements were expressed in parts per billion (ppb). Calibrations were performed at the beginning of the study and then checked daily. Exhalations were repeated until the performance of three values that varied < 10%[16]. Subjects avoided eating, drinking, smoking, and/or exercising for at least 1 hour before testing. FENO measurements were expressed in ppb and as a percentage of the predicted value according to Olin and colleagues[17]. They used height, weight, age, gender, tobacco status, atopy based on total amount of IgE, physician-diagnosed asthma, asthma symptoms during the previous month and reported use of inhaled steroids. We used the same factors excepted for atopy, replaced by self-reported history of physician-diagnosed allergies. No subject needed corticosteroid treatment or had had a crisis in the previous month. Measurements were taken before spirometry and methacholine bronchial challenge (MBC) test.

### Pulmonary function and airway responsiveness

Spirometry was carried out using an electronic spirometer (SpiroStar DXMedikro, L21, St-Germain-en-Laye, France). Forced Vital Capacity (FVC), forced expiratory volume in one second (FEV1), and maximal expiratory flows at various lung volumes were obtained according to ATS recommendations[18]. Results are presented as the ratio or the difference between the observed and predicted values. Airway responsiveness to methacholine was determined using a technique in accordance with published guidelines[19] in an abbreviated version[20]. Only three cumulative doses of methacholine (0.5, 2.5, 7.5 μmol) were administered in sequence using a nebulizer (Mediprom FDC88, Paris, France) delivering doses of 0.5 μmol of methacholine per breath. The system is equipped with a nebulizer De Vilbiss delivering particles 3 μm in diameter. Spirometry was performed just before and three minutes after the inhalations. The test was discontinued either after the inhalation of the last dose or if the FEV1 fell by ≥ 20% below the baseline value, defining a positive MBC test (MBC+). A linear dose-response slope (DRS) was calculated as the percentage decrease in FEV1 at last dose divided by the total administered dose [21]. In order to apply the multiple regression analysis to the DRS, the data was normalized. This normalized dose-response slope (NDRS) transformation (1/(%decrease in FEV1/methacholine μmol +2.5)) had been found to be optimal in a large unexposed population [22]; greater values of NDRS indicating lower AHR.

### Statistical analysis

Statistical analysis was carried out using the Stata package (Stata, College Station, TX, USA). FENO (in ppb and in percentage of the predicted value) was expressed as median and quartiles. Logistic regression analysis was used to assess the relation between MBC+ and FENO adjusting on age, gender, atopy, and FEV1. A value of p < 0.05 was considered significant. Furthermore, the association between NDRS and log-transformed values of FENO was tested in a multiple linear regression model adjusting on the same variables as in the logistic regression analysis.

## Results

Forty-eight lifeguards were invited; 44 subjects (32 men, 12 women) agreed to participate (rate of participation: 92%). One subject refused to perform the MBC test while the curves produced by three subjects were unacceptable. One subject had infectious rhinitis. Therefore, 39 participants (29 men; 10 women) were included in the analyses.

The general baseline characteristics and features of respiratory function, airway responsiveness, and symptoms are shown in Table 1. There were two childhood asthmatics who had not suffered a crisis since adolescence and two adult-onset asthmatics, not having a crisis at the time of testing and not receiving corticosteroid treatment. One of the former and the two latter were classified as having a positive MBC test (MBC+).

**Table 1:** Characteristics of lifeguards

	Males (n = 29)	Females (n = 10)
Age (yr, mean (sd))	35.9 (8.8)	33.3 (10.0)

Height (cm, mean (sd))	180.4 (7.6)	168.3 (7.7)

Smokers (n, (%))	9 (31)	4 (40)
Former smokers (n, (%))	5 (17)	1 (10)
Non-smokers (n, (%)	15 (52)	5 (50)

FEV1		
(L, mean (sd))	5.09 (0.69)	3.83 (0.47)
(% predicted, mean (sd))	121.0 (15.7)	119.7 (11.3)

FVC		
(L, mean (sd))	6.34 (0.69)	4.53 (0.48)
(% predicted, mean (sd))	124.4 (11.5)	123.2 (10.9)

FEV1/FVC		
(% observed, mean (sd))	80.3 (6.1)	84.4 (4.7)
(% predicted, mean (sd))	99.5 (8.0)	102.0 (4.9)

Airway responsiveness		
MBC+ (n, (%))	11 (37.9)	4 (40.0)
NDRS (mean (sd))	0.22 (0.09)	0.19 (0.09)

Personal history of allergy ((n, (%))	2 (6.9)	3 (30.0)

Acute work-related symptoms (n, (%))		
Ocular	21 (72.4)	7 (70.0)
Nasal	15 (51.7)	5 (50.0)
Laryngeal	14 (48.3)	3 (30.0)

FEV1: Forced Expiratory Volume in one Second; FVC: Forced Vital Capacity

MBC: Methacholine Bronchial Challenge test

MBC+: decrease of 20% or more below the baseline value of FEV1 in a methacholine bronchial challenge test.

NDRS: Normalized Dose Response Slope

The proportion of current smokers was greater among females (40%) than among males (31%); since the number of ex-smokers was so small, we chose to classify them as non-smokers. Overall, pulmonary function values exceeded the predicted ones both among males (121%) and females (119%) for the FEV1. Two men (2/29 = 6.9%) and three women (3/10 = 30.0%) reported a personal history of allergy. No cases of chronic bronchitis or dyspnea were recorded. However, there was a high prevalence of acute, irritant symptoms both among males and females, with prevalence rates ranging from 30.0% for laryngeal irritation among females to 72.4% for ocular symptoms among males.

Of the 39 lifeguards, 15 (38.5%) were classified as having MBC+. From these, 11 were males and four were females, thus giving prevalence rates of 37.9% and 40.0% respectively.

FENO for the whole group was not normally distributed. The median FENO values for reactors and non-reactors stratified by sex and smoking status are shown in Table 2.

**Table 2:** FENO (median [percentile 25, percentile 75] in ppb and as % predicted) in reactors and non-reactors stratified by sex and smoking status

		MBC -		MBC+
	
		FENO		FENO
	n	ppb	n	ppb
		(%pred)		(%pred)
All	24	12.5 [8.2, 17.3]	15	18.9 [11.9, 36.3]
		(73.5 [44.2, 96.5])		(90.1 [59.6, 219.9])

Males	18	13.7 [8.7, 19.2]	11	22.4 [15.4, 36.3]
		(73.5 [44.3, 99.0])		(118.2 [77.7, 219.9])
Smokers	6	9.8 [7.3, 14.8]	3	17.1 [7.6, 30.1]
		(64.5 (44.0,103.7])		(131.8 [55.5, 219.9])
Non-smokers	12*	14.8 [9.9, 19.8]	8**	26.1 [17.1, 45.9]
		(78.0 [48.6, 96.5))		(104.2 [78.4, 199.7])

Females	6	9.6 [7.1, 13.8]	4	11.5 [10.8, 37.2]
		(71.3 [39.9, 93.6])		(65.6 [54.8, 177.5])
Smokers	3	13.8 [10.1, 24.5]	1°	11.1
		(93.6 [88.8,196.8])		(71.5)
Non-smokers	3	7.1 [4.8, 9.1]	3°°	11.9 [10.5, 62.5]
		(39.9 [25.1, 53.7])		(59.6 [49.9, 283.6])

* one with a history of allergies FENO = 7.6 ppb (24.9% pred)

** one with a history of allergies FENO = 36.3 ppb (118.2% pred)

° one with a history of allergies

°° two with a history of allergies FENO = 10.5 ppb (49.9% pred) and 62.5 (283.6% pred)

The median FENO in reactors was 18.9 ppb (11.9 to 36.3 ppb; 59.6 to 219.9% predicted), whereas in non-reactors it was 12.5 ppb (8.2 to 17.3 ppb; 44.2 to 96.5% predicted). A similar trend toward greater FENO values among reactors was noticed across all male subgroups but less so among females; however, the latter subgroups were too small for comparison.

**Figure 1:**
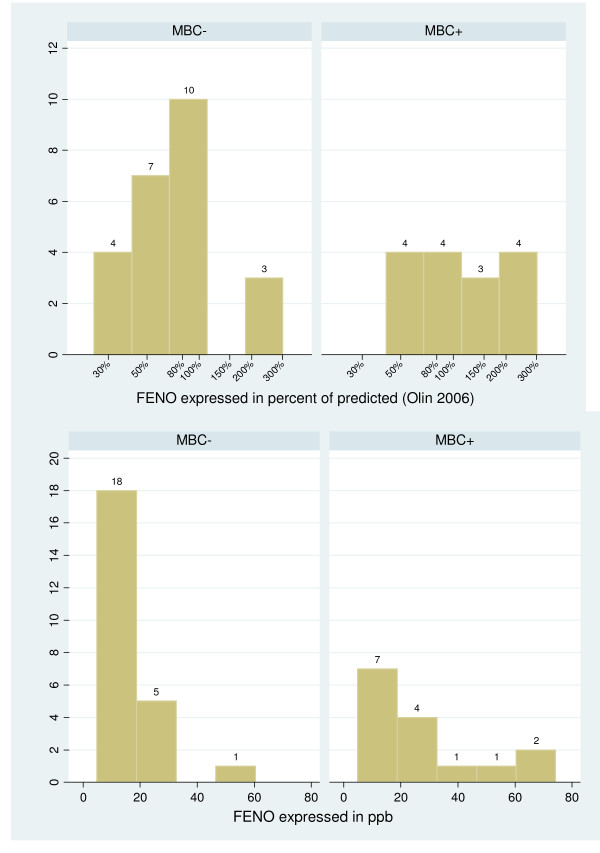
**Distribution of measured FENO in reactors (MBC+) and non-reactors (MBC-)**.

The distribution of FENO in reactors and non-reactors is shown in Figure 1 (FENO in percent predicted and FENO in ppb). The distribution of lifeguards according to the arbitrary FENO cutoff point of 60% of the predicted value, and according to MBC+ or MBC- groups was as follows: FENO ≥ 60% (11 MBC+/14 MBC-); FENO < 60% pred.: (4 MBC+/10 MBC-). The sensitivity was 80%, the specificity 42%, the positive predictive value 44% and the negative predictive value 71%. FENO had an effect on AHR adjusting or not on atopy, smoking, and FEV1 in the logistic regression model. Similarly, none of these factors had a significant effect on AHR when adjusting on FENO.

**Figure 2:**
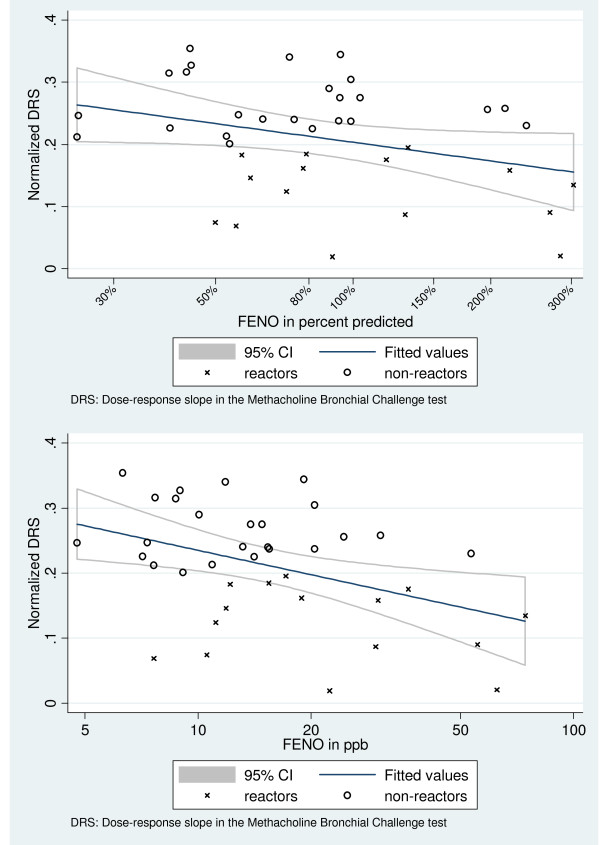
**Association between Normalized Dose Response Slope and measured FENO in reactors (MBC+) and non-reactors (MBC-)**.

Reactors tended to have greater FENO values and smaller NDRS values than non-reactors (Figure 2). In a multiple linear regression model predicting NDRS adjusted on sex, smoking, FEV1 (difference between observed and predicted values) and atopy (Table 3), log FENO was a significant predictor (p = 0.01) as was atopy (p = 0.02).

**Table 3:** Multiple linear regression models of normalized dose response slope according to log FENO in percent predicted, difference between observed and predicted FEV1, sex, smoking status, and personal history of allergies.

	Normalized dose response slope		
	coefficient	95% CI	p
Log FENO (% predicted)	-0.119	[-0.210;-0.029]	0.011
Difference between observed and predicted FEV1	0.009	[-0.012;0.030]	0.373
Sex			
Male versus female	0.025	[-0.035;0.085]	0.399
Smoking status			
Smokers versus non smokers	0.052	[-0.001;0.105]	0.056
Personal history of allergies versus no personal history	-0.090	[-0.168;-0.013]	0.024

95% CI: 95% confidence interval

## Discussion

This study showed that the concentration of FENO in lifeguards working in indoor swimming pools correlates significantly with the degree of airway responsiveness measured with methacholine; this relationship was not affected by smoking status, gender, or self-reported history of allergies. Furthermore, the 60% of predicted value cutoff-point for "abnormal" FENO was sensitive in discriminating reactors from non-reactors. To our knowledge, no similar data have been collected previously in working populations.

There was a high prevalence of AHR among male (37.9%) and female (40.0%) lifeguards. These prevalence rates are higher than but comparable with the corresponding rates (13.7% males; 28.2% females) reported previously in another population of indoor lifeguards[15]. The clinical significance of AHR in our subjects is not straightforward as the population contains only two prevalent asthmatics. Indeed, although AHR is a common feature of asthma, dissociation between AHR and inflammation has been documented[23] so the exact mechanism by which inflammation causes AHR is unknown.

FENO levels are correlated with eosinophilic airway inflammation in asthmatics and the test has been proposed for asthma-screening in young adults[24]. However, the significance of elevated FENO in workers without overt asthma remains unclear. The few published studies on this topic found high FENO levels in workers exposed both to sensitizers and irritants[8-10], a finding suggesting that eosinophilic inflammation might not be the only mechanism to explain the increased NO production in workers. Incidentally, studies of endurance athletes, - a population closer to our lifeguards than asthmatics -, did not find a correlation between increased FENO and eosinophil counts in induced sputum[25].

Bearing in mind the above considerations and the known mechanisms of NO production[26], we are tempted to speculate that the raised FENO levels in reactors could be due to sub-clinical airway inflammation caused by exposure to the highly irritant chloramine NCl3. The extent to which such inflammation is eosinophilic in nature or not is irrelevant since exposure to pollutants can raise NO production by eliciting changes in oxide synthase but also though oxidative stress[10,27]. In addition, an increase in the permeability of airway epithelium to allergens due to chloramines has been described in lifeguards[27] and could have played a role in our subjects, although, in this case, we would have expected a better correlation with atopy. Whether this high prevalence of AHR and concomitant high levels of FENO is however due to any specific exposure is beyond the scope of this paper.

Atopy is important in the relationship between FENO and AHR. Verges and colleagues[25] showed that endurance athletes with AHR had significantly higher FENO levels and were more frequently atopic than subjects without AHR. Franklin and colleagues[28] showed elevated FENO in atopic children AHR+ but not in atopic children AHR-. Subsequently, the same team showed similar findings in the parents of these children, with an intriguing negative association between FENO and DRS in non-atopic subjects and a positive association in the atopic ones, asthma being not directly related to levels of FENO once the interaction FENO atopy was accounted for[14]. To the extent that a personal history of allergies can be equated with atopy, our results are at variance with these data. While we acknowledge that the number of our workers reporting allergies was small, we must stress that this parameter is more closely related to the risk of work related symptoms over time than to atopy based on a skin prick test[29]. Furthermore, personal history of allergy has been reported to be as efficient as positive skin prick tests to common allergens in detecting associations between atopic diathesis and allergic respiratory diseases in working populations, including animal handlers, bakers, and workers exposed to latex[29]. However, it is possible that undetected atopy could explain part of the association between FENO and AHR. However, given that this is the second population of lifeguards in which we observed a high prevalence of AHR, it is highly unlikely that this finding is due to atopy alone.

We used 60% of the predicted values of Olin and colleagues[17] as a cutoff-point for "abnormal" FENO for three reasons. First, current guidelines do not yet specify "normal" values and evidence is accumulating that the "optimal" cutoff point for screening for asthma will not necessarily be suitable for other populations. Second, unlike others, the equations proposed by Olin and colleagues[17] take into account some of the most known confounding factors namely atopy, gender, and smoking status. Finally, given the preliminary character of this study, a rough threshold -- chosen by visual inspection of the distribution of FENO (Figure 1) -- was enough for us to test our strategy.

One strength of this study was the quality of data collection. Our team has been measuring airway responsiveness for almost two decades and our technique is well standardized. In addition, FENO measurements are simple to perform and were carried out according to current guidelines at constant expiratory flows of 50 mL.s^-1 ^and systematically before spirometry and methacholine challenge. In this respect, the differences we observed between smokers versus non-smokers (Table 2) is in agreement with recent studies[17] including our own[7]. Concerning limitations, our sample size was indeed small but represented recruitment from *all *indoor pools in our region. Notwithstanding, we were able to document a significant relationship between FENO levels and two indices of AHR in apparently healthy lifeguards, which was the main reason for this preliminary study. In this context, a non-exposed, control group would have added little to this evidence.

## Conclusions

In conclusion, our results suggest that FENO measurements are potentially useful in detecting workers with AHR considered as a risk factor for the development of symptoms. Using a less than optimal cutoff-point for "abnormal" FENO, we showed that high FENO values are associated with AHR while low FENO values tended to be associated with normal airway responsiveness. Further prospective longitudinal studies exploring the relationship between FENO and AHR are necessary to improve our knowledge of the significance of FENO in working populations.

## Competing interests

The authors declare that they have no competing interests.

## Authors' contributions

VD made a substantial contribution to the analysis of data and was involved in drafting the manuscript. AB made a substantial contribution to interpretation of data, and made important criticism on the manuscript intellectual content. NM performed data-acquisition and was involved in drafting the manuscript. PW performed data-analysis and interpretation and was involved in drafting the manuscript. All authors read and approved the final manuscript.

## Pre-publication history

The pre-publication history for this paper can be accessed here:

http://www.biomedcentral.com/1471-2466/9/53/prepub
